# Ligand-Tuned Multi-Color Luminescence of Single Aluminum (III) Ion Atomic Centers and Their Selective Sensitivity to Different Metal Ions

**DOI:** 10.3390/ma15155199

**Published:** 2022-07-27

**Authors:** Qian Wang, Longlong Li, Qinglin Tang, Jin Liu, Yao Wang, Jiuxing Wang, Matt J. Kipper, Haijiao Xie, Laurence A. Belfiore, Jianguo Tang

**Affiliations:** 1 Institute of Hybrid Materials, National Center of International Joint Research for Hybrid Materials Technology, National Base of International Sci. & Tech. Cooperation on Hybrid Materials, Qingdao University, 308 Ningxia Road, Qingdao 266071, China; reamiss@163.com (Q.W.); babylove8856@126.com (L.L.); a15666920912@163.com (Q.T.); liujin0620@126.com (J.L.); wangyaoqdu@126.com (Y.W.); jiuxingwang@qdu.edu.cn (J.W.); belfiore@engr.colostate.edu (L.A.B.); 2 Department of Chemical and Biological Engineering, Colorado State University, Fort Collins, CO 80523, USA; matthew.kipper@colostate.edu; 3 Hangzhou Yanqu Information Technology Co., Ltd., Y2, 2nd Floor, Building 2, Xixi Legu Creative Pioneering Park, No. 712 Wen’er West Road, Xihu District, Hangzhou 310003, China; xiehaijiao@shiyanjia.com

**Keywords:** aluminum complex, ligand tuned, CLM, sensitivity

## Abstract

Achieving multi-color luminescence with a single atomic center in transition metal complexes is a challenge. In this work, luminescent materials with tunable emission properties were realized by complexation between aluminum (III) ions with the ligands 3-hydroxyflavone (3-HF) and 5,7-dichloro-8-hydroxyquinoline (DCHQ). Aluminum (III) complexes with a single ligand emitted blue from 3-HF and green from DCHQ. High quantum yields (QYs) of 29.42% and 37.00% were also obtained, respectively. DFT calculations revealed details of the photophysical properties of the complexes. Correspondingly, cyan light emission was obtained if these two complexes were mixed together, from which the emission wavelength was located at 470 nm and the QY was 20.52%, under 290 nm excitation. More importantly, the cyan light emitted by the mixtures had selective sensitivity to different metal ions, resulting in either quenching the fluorescence (in the case of Fe^3+^) or enhancing the fluorescence (in the case of In^3+^). The fluorescence enhancement effect of In^3+^ on metal complexes has not been previously reported, neither for transition metal nor lanthanide ions. The linear quenching behavior of Fe^3+^ functions in the 50–700 μM concentration range, and the linear enhancement behavior of In^3+^ is demonstrated in the 300–800 mM concentration range.

## 1. Introduction

It is unusual that a single atomic center in transition metal complexes can be tuned to emit multi-color luminescence, even when different ligands are used, because the energy levels of outer electron orbitals in transition metal ions are fixed [1,2]. However, our findings on the fluorescence of aluminum ion complexes defy this trend. There are few publications that report the aluminum luminescence phenomenon, and they report single-emission behavior [3]. Investigations on aluminum luminescence behavior are interesting for new light resources in light-emitting diodes (LEDs), photon energy conversions, and applications for agriculture, environmental monitoring, and bioanalytical devices. The aluminum ion is also an important ion in biological systems [4].

Aluminum is usually deemed the most abundant metallic element in nature [5,6,7,8]. Aluminum rarely exists in its elemental form, because of its ground-state electronic configuration [Ne] 3s^2^3p^1^, which easily loses the three electrons in the outer orbitals. Instead, aluminum usually occurs in the +3 oxidation state, with completely vacant 3s and 3p electron orbitals [9,10]. The vacant orbitals impart the ability to accept electrons from ligands and thereby perturb ligand luminescence. Organic ligand–aluminum complexes, including tris (8-hydroxyquinoline) aluminum, flavonoids, and Schiff base complexes, have been reported [11,12]. Tang et al. first found that aluminum could complex with 8-hydroxyquinoline to synthesize fluorescent materials [13]. While 8-hydroxyquinoline exhibits weak emission of blue fluorescence, the 8-hydroxyquinoline–aluminum complex can emit strong green fluorescence [14]; the complexation improves the emission intensity and also results in a red shift of the emission wavelength.

Tuning multi-color luminescence using a single-metal ion is challenging since multi-color tuning is normally achieved using different metal ion centers and changing the ratio of different components to emit different-wavelength fluorescence. For example, Wang et al. used lanthanide complexes to tune high-efficiency white light emission [15,16]. In addition, there was a publication to show a relatively novel method to observe the behavior of multi-color electrochromism of nanosheets by changing the applied voltage [17]. Aluminum is an ideal candidate to achieve multi-color luminescence from a single-metal center, because of its special outer electron orbital configuration. Cyan light is an indispensable energy source for plant growth [18], but materials emitting cyan light are rare in nature. Currently, cyan light is usually obtained from the preparation of phosphors to fill the cyan gap of organic light-emitting diodes (OLEDs), which is a cumbersome process [19,20]. In particular, research about cyan emission materials from aluminum (III) atomic centers has not been widely reported. Herein, we hypothesize that cyan light can be obtained through the combination of blue and green light by using different ligands complexed with an aluminum (III) ion metal center.

In this work, a method for synthesizing cyan luminescent material (CLM) with single-ion aluminum (III) ion centers is proposed. The organic ligand 3-hydroxyflavone (3-HF) itself has no fluorescence, and 5,7-dichloro-8-hydroxyquinoline (DCHQ) has extremely weak fluorescence. 3-HF and DCHQ were chosen as ligands to prepare blue-emitting and green-emitting aluminum complexes, with emission peaks at 460 nm and 512 nm, respectively. After mixing solutions of the two complexes in different volume ratios, a bright cyan emission from single aluminum (III) ion centers was achieved by adjusting the ratio of the two complexes. In addition, the cyan luminescent solution (CLS) has selective sensitivity to metal ions, such as Fe^3+^ and In^3+^. It is foreseeable that CLM can be used not only as a sensor for various metal ions but also for the preparation of high-intensity CLM from double-metal-ion complexes.

## 2. Experimental Section

### 2.1. Materials

3-Hydroxyflavone (3-HF) was procured from Alfa Aesar (Shanghai, China). Iron chloride tetrahydrate (FeCl_2_·4H_2_O), 4-hydroxy-1,5-naphthyridine (ND), and 4,4′-bipyridine (4,4′-Bpy) were procured from Shanghai Macklin Biochemical Co., Ltd. (China). Europium chloride hexahydrate (EuCl_3_·6H_2_O), terbium chloride hexahydrate (TbCl_3_·6H_2_O), and 8-hydroxyquinoline-5-sulfonic acid (HQSA) were procured from Aladdin Industrial Corporation (Shanghai, China). 5,7-Dichloro-8-hydroxyquinoline (DCHQ), dichloromethane, absolute ethanol, and all the other metal salts (CuCl_2_·2H_2_O, CaCl_2_, FeCl_3_·6H_2_O, CoCl_2_·6H_2_O, ZnCl_3_, MgCl_2_, CrCl_3_·6H_2_O, NiCl_2_·6H_2_O, MnCl_2_·4H_2_O, LiCl, InCl_3_) were purchased from Sinopharm Chemical Reagent Co., Ltd. (Beijing, China). Al(NO_3_)_3_·9H_2_O and 6-hydroxy-1-tetralone (6-HT) were procured from Adamas-beta Titan Scientific Co., Ltd. (Shanghai, China). All the chemicals used were of analytical grade and without further purification.

### 2.2. Synthesis

**Figure 1:**
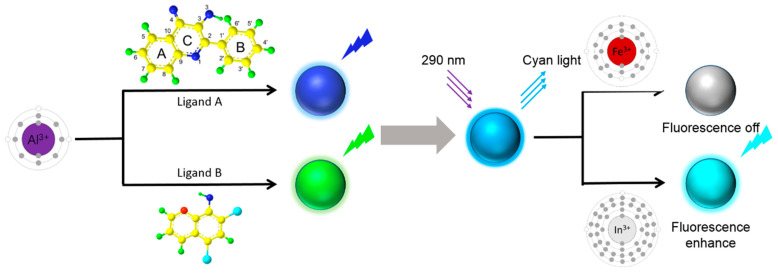
Schematic diagram of the method for tuning the color of cyan light by the combination of blue light and green light, and the sensitivity of CLM to metal ions with both enhancement and quenching effects.

**Synthesis ofAl(3-HF)_2_.** A solution of aluminum (III) ions (0.0010 M) was prepared by adding Al(NO_3_)_3_·9H_2_O crystals to absolute ethanol and stirring for half an hour. Meanwhile, 3-HF (0.0020 M) was dissolved in absolute ethanol and stirred for half an hour to obtain an almost colorless clear solution. Then, the solution of Al(3-HF)_2_ was prepared by adding a solution of aluminum (III) ion to the same volume of a solution of 3-HF under continuous stirring. Upon addition of aluminum (III) ions, the solution color changed from almost colorless to faint yellow. The solution of Al(3-HF)_2_ was prepared again, as described before, to characterize the structure of the complex and to obtain a solid powder. Next, it was poured into an evaporating dish and placed in a clean fume hood for 72 h. Finally, the wet sample was dried using a vacuum-drying oven. The chemical structure of 3-HF is shown in Figure S1 and Figure 1.

**Synthesis of Al(DCHQ)_3_.** The solution of aluminum (III) ions (0.0010 M) was prepared, as described before. DCHQ (0.0030 M) was suspended in dichloromethane to obtain an off-white suspension. When the aluminum solution and DCHQ suspension were combined, the mixture changed from off-white to clear yellow. Likewise, the solid powder of Al(DCHQ)_3_ was obtained in the same way as that of Al(3-HF)_2_. The chemical structures of DCHQ and Al(DCHQ)_3_ are also shown in Figure S1 and Figure 1, respectively.

**Cyan Emission Tuning.** The solutions of Al(3-HF)_2_ and Al(DCHQ)_3_ were mixed in different volume ratios, stirred for 2 h, and then stored stationary at room temperature for 30 min. The fluorescence spectral data obtained by measuring these solutions were exported into the CIE coordinates and were compared to the standard cyan light area. Then, the ratio of the two components was fine-tuned by adjusting the ratio of aluminum (III) ions to the two ligands to get the final emission peak of the mixed solution close to 470 nm. The process of tuning the emitted light color is illustrated in Figure 1.

### 2.3. Characterization

The morphological structures of the complexes were characterized using TEM (JEM-F2100, JEOJ, Kyoto, Japan), with the electron microscope operating at 100 kV. The FT-IR spectra of the two ligands and complexes were recorded using an FT-IR spectrometer (Nicolet 5700, Thermo Nicolet Corporation, Waltham, MA, USA). XPS measurements were performed using a Thermo Escalab XPS instrument (ESCALAB 250XI, Thermo Fischer Scientific, Waltham, MA, USA). UV–VIS absorption data were obtained using a UV–VIS spectrophotometer (Lambda 750 S). The fluorescence spectra, lifetimes, and quantum yields were recorded using a steady-state transient photoluminescence spectrometer (FLS1000, Edinburgh Instruments, Edinburgh, UK).

### 2.4. Smart Sensing to Different Metal Ions

**Detection of Fe^3+^ Content.** Fe^3+^ detection was performed by adding the same volume of CLS (2 mL) to solutions containing different Fe^3+^ concentrations (50, 100, 150, 200, 250, 300, 350, 400, 450, 500, 550, 600, 650, and 700 μM). After addition of the aluminum complexes, the test solutions were kept at room temperature for 10 min; the emission spectra were then measured under 290 nm light excitation.

**Detection of In^3+^ Content.** Likewise, 2 mL solutions of In^3+^ with different concentrations (250, 300, 350, 400, 450, 500, 550, 600, 650, 700, 750, 800, 850, 900 mM) were prepared. The test solutions were prepared by the same method as for the detection of Fe^3+^. The emission spectra of the solutions were obtained under 290 nm light excitation.

**Selective analysis of ions.** To investigate the selectivity of CLS for metal chloride salts, stock solutions of each ion (0.0100 M) were prepared in absolute ethanol. An equal volume of CLS was added to each test sample and allowed to stand at room temperature for 10 min. Finally, the florescence spectra of the mixed solutions were measured.

### 2.5. Density Functional Theory (DFT) Calculations

The ground-state geometry of Al(3-HF)_2_ and Al(DCHQ)_3_ was optimized using DFT calculations, and the excited states were calculated with time-dependent DFT (TDDFT) at the optimized ground-state geometry. All calculations were performed using the hybrid B3LYP functional and the 6-311G* basis set. Hole–electron analysis for S1 and T1 transitions was performed using Multiwfn.

## 3. Results and Discussion

### 3.1. Complex Structure of Different Organic Ligands

**Figure 2:**
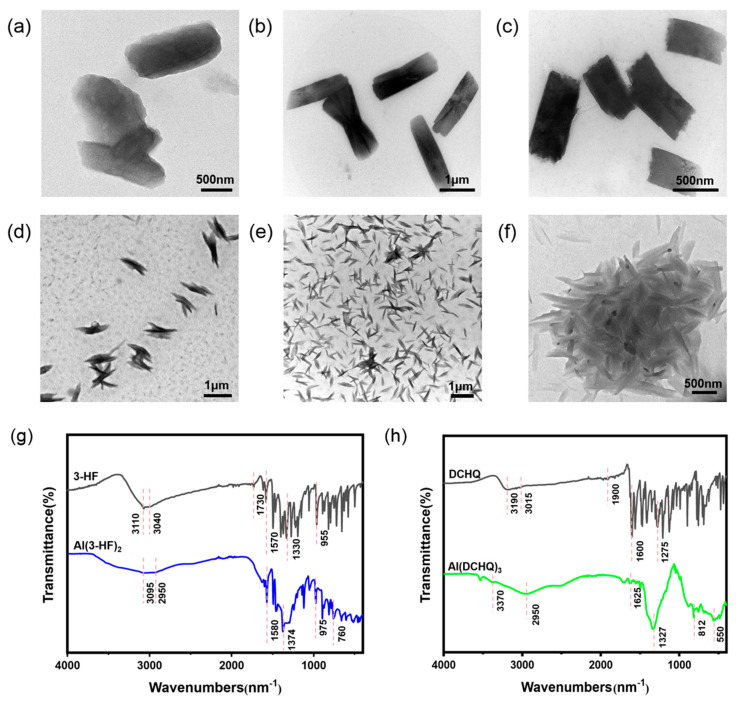
TEM images of different concentrations of (**a**–**c**) Al(3-HF)_2_; the concentrations of aluminum (III) ions are (**a**) 0.0005 M, (**b**) 0.0010 M, and (**c**) 0.0020 M. TEM images of different concentrations of (**d**–**f**) Al(DCHQ)_3_; the concentrations of aluminum (III) ions are (**d**) 0.0005 M, (**e**) 0.0010 M, and (**f**) 0.0020 M. FT-IR spectra of (**g**) 3-HF and Al(3-HF)_2_ and of (**h**) DCHQ and Al(DCHQ)_3_.

The morphological structures of Al(3-HF)_2_ and Al(DCHQ)_3_ were characterized by transmission electron microscopy (TEM). As can be seen in Figure 2, the morphologies of the complexes changed as the concentration of aluminum (III) ions increased. The structures of Al(3-HF)_2_ are shown in Figure 2a–c. When the concentration of aluminum (III) ions was 0.0005 M, the complex showed a fuzzy sheet structure. With increasing concentration of aluminum (III) ions, the complex showed good dispersity and regular rod-like morphology. Until the concentration of aluminum (III) ions reached 0.0020 M, the complexes were stacked. The morphologies of Al(DCHQ)_3_ are shown in Figure 2d–f, with the influence of concentration changes in aluminum (III) ions. These images imply that the optimal concentration of Al(DCHQ)_3_ should be 0.001 M (for aluminum (III) ion) as it shows uniformly dispersed and non-aggregated particles.

The FT-IR spectra of aluminum complexes and corresponding ligands are shown in Figure 2g,h, which provide binding and structural information. In the IR spectrum of free 3-HF in Figure 2g, bands at 3110 and 3040 cm^−1^ were observed, which represent the stretching vibration of O–H and C–H. The shoulders positioned at 1730 and 1570 cm^−1^ were assigned to the stretching modes of C=O and C=C, while the bands at 1330 and 955 cm^−1^ were attributed to the C–O stretching and C–H bending vibration [21]. For Al(3-HF)_2_, the band at 3095 cm^−1^ indicated weakened stretching vibration of O–H due to complexation, while the band at 2950 cm^−1^ represented C–H stretching. The band at 1730 cm^−1^ disappeared due to complexation [22]. In addition, the bands located at 1580 and 1374 cm^−1^ indicated the stretching modes of C=C and C-O, while the band located at 975 cm^−1^ indicated the C–H bending vibration. Notably, the new band positioned at 760 cm^−1^ could be attributed to Al–O stretching. These findings confirm the complexation between aluminum (III) ions and 3-HF.

In the IR spectrum of free DCHQ in Figure 2h, the broad characteristic peak positioned at 3190 cm^−1^ was due to the O–H stretching vibration, while the band at 3015 cm^−1^ was attributed to C–H stretching vibration in an aromatic ring. The band at 1900 cm^−1^ indicated C=N vibration. The peaks at 1600 and 1275 cm^−1^ were attributed to the stretching modes of C=C and C–O [23]. Upon complexation, the Al(DCHQ)_3_ spectrum had a reduced band at 3370 cm^−1^, which could be assigned to complexation. The band at 2950 cm^−1^ represented the C–H stretching vibration, which is consistent with the results for the free ligand. The band at 1900 cm^−1^ in the spectrum of DCHQ disappeared due to complexation. At the same time, the peaks at 1625 and 1327 cm^−1^ could be attributed to the stretching of C=C and C–O. Notably, the enhancement of the peak at 812 cm^−1^ corresponded to the vibration of Al-O, indicating that the coordination of Al-O is relatively strong [24]. Furthermore, the band positioned at 550 cm^−1^ represented the Al–N vibrational mode [25].

**Figure 3:**
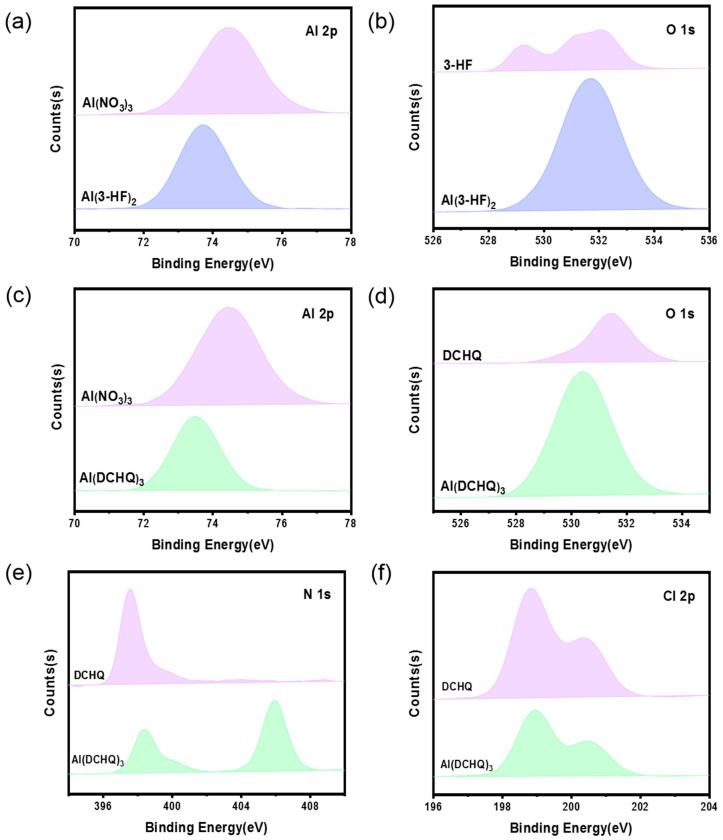
XPS spectra of (**a**) Al 2p in Al(NO_3_)_3_ and Al(3-HF)_2_, (**b**) O 1s in 3-HF and Al(3-HF)_2_, (**c**) Al 2p in Al(NO_3_)_3_ and Al(DCHQ)_3_, (**d**) O 1s in DCHQ and Al(DCHQ)_3_, (**e**) N 1s in DCHQ and Al(DCHQ)_3_, and (**f**) Cl 2p in DCHQ and Al(DCHQ)_3_.

X-ray photoelectron spectroscopy (XPS) provided information about metal and ligand interactions in the synthesized complexes. The full XPS survey data of the two ligands and complexes are provided in Figure S2, and these confirm the existence of characteristic components in Al(3-HF)_2_ and Al(DCHQ)_3_, respectively. The core electronic binding energies of Al 2p, O 1s, N 1s, and Cl 2p for Al(NO_3_)_3_ ligands and complexes are compared in Figure 3. There was a certain difference in the binding energy of Al 2p between Al(3-HF)_2_ and the metal salt. Compared to Al(NO_3_)_3_, the binding energy of Al 2p in Al(3-HF)_2_ decreased by 1.1 eV (i.e., 74.8 vs. 73.7 eV), as shown in Figure 3a. Similarly, the binding energy of Al 2p in Al(DCHQ)_3_ shifted by 1.4 eV (i.e., 74.8 vs. 73.4 eV), as shown in Figure 3c. As for the difference in the binding energy of O 1s between the ligand 3-HF (532.15 eV) and Al(3-HF)_2_ (531.7 eV), the shift of binding energy was 0.45 eV before and after complex formation (Figure 3c), while the shift between DCHQ (530.45 eV) and Al(DCHQ)_3_ (531.45 eV) was 1 eV (Figure 3d). In contrast, the N 1s binding energy increased (i.e., 397.6 vs. 405.9 eV) in Al(DCHQ)_3_ (Figure 3e) compared to Al(NO_3_)_3_, which is caused by the Al–N coordination probably due to N-bond distortion [25]. The binding energy of Cl 2p had no shift in DCHQ and Al(DCHQ)_3_ (Figure 3f), and the binding energy of C 1s had no shift in either 3-HF and Al(3-HF)_2_ or DCHQ and Al(DCHQ)_3_, as shown in Figure S3.

### 3.2. Photophysical Properties of Al(3-HF)_2_ and Al(DCHQ)_3_

**Figure 4:**
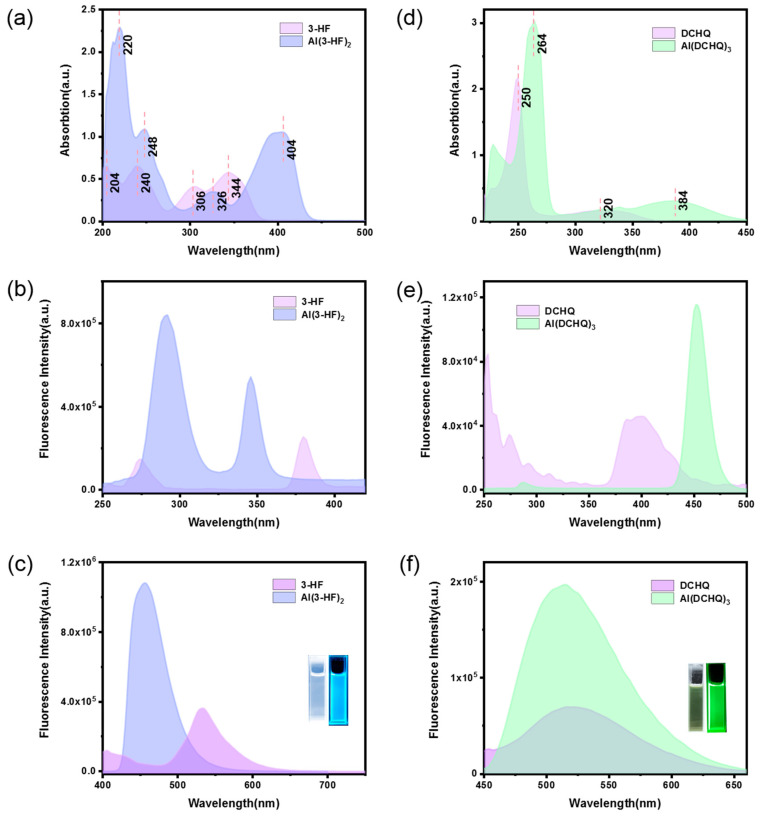
Comparison of (**a**) UV–VIS absorbance spectra, (**b**) excitation spectra, and (**c**) fluorescence intensity of 3-HF and Al(3-HF)_2_. Comparison of (**d**) UV–VIS absorbance spectra, (**e**) excitation spectra, and (**f**) fluorescence intensity of DCHQ and Al(DCHQ)_3_.

The photophysical properties of Al(3-HF)_2_ were significantly different compared to 3-HF. As shown in Figure 4c, the solution of Al(3-HF)_2_ (shown in the inset picture) was lighter yellow under sunlight and emitted a bright-blue light under irradiation with ultraviolet light. This indicates that Al(3-HF)_2_ exhibits photoluminescence. The UV–VIS absorption spectra of 3-HF and Al(3-HF)_2_ in ethanol solutions are presented in Figure 4a. Due to π–π* electron transition, the UV–VIS spectra of 3-HF and Al(3-HF)_2_ had their characteristic absorption [26], in which the UV–VIS spectrum of 3-HF showed two absorption bands of 306–344 nm and 204–240 nm [27]. Among them, the largest absorption peak was located at 344 nm [26]. Since the coordination of 3-HF with aluminum (III) ions occurs at positions 3 and 4 of ring C (Figure 1), after the formation of the aluminum complex, the absorption bands red-shifted to 220–248 nm and 326–404 nm, respectively.

Figure 4b,c shows the excitation and emission spectra of 3-HF and Al(3-HF)_2_. In Figure 4c, 3-HF shows weak fluorescence at 535 nm when excited at 380 nm. The maximum excitation and emission wavelengths of Al(3-HF)_2_ were 290 nm and 460 nm, respectively, which confirms that the fluorescence emission of Al(3-HF)_2_ is in the blue light range. Thus, we can find that the excitation wavelength of Al(3-HF)_2_ at 290 nm has a significant blue shift, compared to 3-HF at 346 nm, which is caused by the coordination interaction between 3-HF and aluminum (III) ions [28]. In this work, two other organic ligands (4-hydroxy-1,5-naphthyridine (ND) and 4-4’-bipyridine (4-4’-Bpy); Figure S1) with excellent performance, were selected, and the corresponding complexes were prepared according to the same preparation process. They can also form complexes with aluminum (III) ions to emit blue fluorescence. The differences in fluorescence intensity and lifetime between Al(3-HF)_2_ and these complexes were investigated. From Figure S4, it can be clearly seen that compared to other complexes, Al(3-HF)_2_ had high fluorescence intensity. Furthermore, the transient fluorescence decay curves of the three complexes are shown in Figure S5 and corresponding parameters are summarized in Table S1. As shown in Figure S5, the decay curves of Al(3-HF)_2_ were well fitted by a single-exponential function, Y = A + B_1_exp(−t/τ_1_), where Y and A represent the emission intensity at time t and 0, respectively; B_1_ is a fitting constants; and τ_1_ is the decay time for the exponential [29]. Based on the fitting data, the fluorescence lifetime of Al(3-HF)_2_ was 5.606 ns and the QY of the Al(3-HF)_2_ was 29.42%. According to the principle of the CIE standard chromaticity system, the coordinate points on the chromaticity diagram are shown in Figure S6.

The optical properties of Al(DCHQ)_3_ are shown in Figure 4d,f. The solution of Al(DCHQ)_3_ was light yellow under sunlight (shown in the inset picture in Figure 4f) and emitted green light under irradiation of ultraviolet light. The UV–VIS absorption spectra of DCHQ and Al(DCHQ)_3_ are shown in Figure 4d. In the UV–VIS spectrum of DCHQ, the short-wave absorption peak at 250 nm corresponded to the π–π* electronic transition of the quinoline ring, while the peak at 320 nm corresponded to the n––π* electronic transition of the N atom of the quinoline ring [30]. In the spectrum of Al(DCHQ)_3_, the absorption band at 264 nm shifted from 250 nm in the DCHQ spectrum. Meanwhile, the absorption peak intensity of Al(DCHQ)_3_ at 264 nm was much stronger than that of DCHQ at 250 nm, which indicates the influence of complexation with aluminum (III) ions in Al(DCHQ)_3_. Furthermore, the n–π* electronic transition at 320 nm was significantly weakened due to the coordination of the N atom to the aluminum (III) ion, which is evidence for the formation of coordination bonds between DCHQ and aluminum (III) ions. Impressively, a new band appeared at 384 nm, which may come from the bonding electronic transition between aluminum (III) ions and DCHQ. That is, it may be caused by the metal-to-ligand charge transfer (MLCT) transition [27].

Figure 4e,f shows the excitation and emission spectra of DCHQ and Al(DCHQ). The maximum excitation and emission wavelengths of Al(DCHQ)_3_ were 452 nm and 512 nm, respectively, which confirms that the fluorescence emission of Al(DCHQ)_3_ is in the green light range. The organic ligand DCHQ emitted weak fluorescence, and Al(DCHQ)_3_ emitted with strong intensity. Furthermore, the fluorescence spectra of the aluminum complexes with other organic ligands (8-hydroxyquinoline-5-sulfonic acid (HQSA), 6-hydroxy-1-tetralone (6-HT); Figure S1) are shown in Figure S7. The complexes of Al-HQSA and Al-6-HT exhibited weak emissions. The fluorescence lifetimes and QYs of different complexes are listed in Table S2. As shown in Figure S8, the fluorescence intensity decay was fitted to a single-exponential decay function, and the calculated fluorescence lifetime of Al(DCHQ)_3_ was 18.810 ns. In addition, Al(DCHQ)_3_ had a high QY of 37%. According to the principle of the CIE standard chromaticity system, the coordinate point of green fluorescence in the chromaticity diagram is shown in Figure S9 [31].

### 3.3. Density Functional Theory Calculation

**Figure 5:**
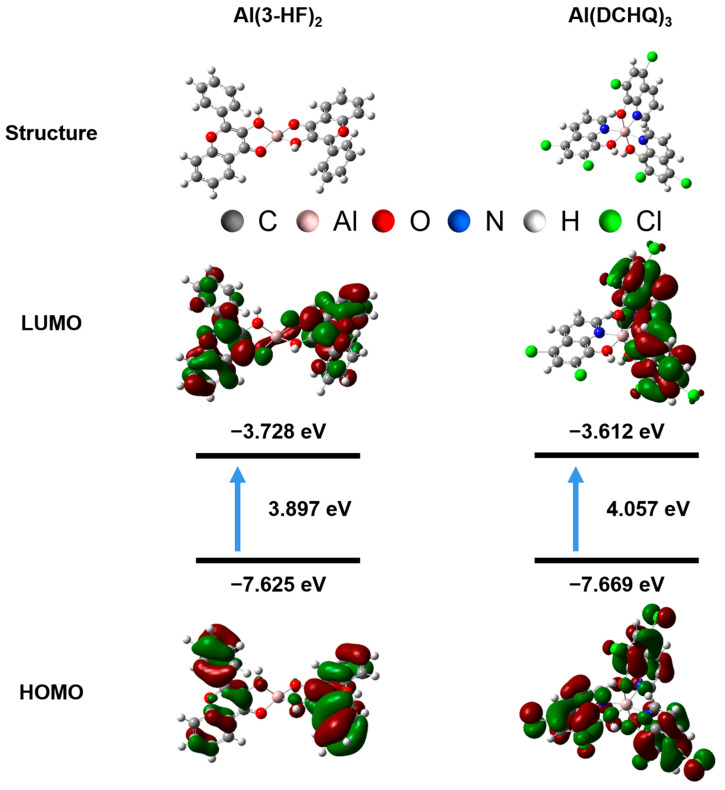
Energy−level diagram containing the HOMO and LUMO for Al(3-HF)_2_ and Al(DCHQ)_3_ obtained from DFT calculations.

The structural details and photophysical properties of the complexes were further studied through density functional theory (DFT) simulation. The optimized structures and frontier molecular orbitals of the complexes are shown in Figure 5. As shown in column A of Figure 5, the highest occupied molecular orbital (HOMO) and the lowest unoccupied molecular orbital (LUMO) of Al(3-HF)_2_ were uniformly distributed in the phenyl ring in 3-HF, with an energy gap of 4.057 eV between the two orbital energy levels, which indicates the occurrence of intra-ligand charge transfer (ILCT) [26]. Likewise, the HOMO of Al(DCHQ)_3_ was spread over the phenyl rings of all ligands, while its LUMO was mainly located on the two ligands. The energy gap between the two orbitals was 3.897 eV, indicating the ligand-to-ligand charge transfer (LLCT) occurred [32]. In addition, the lowest excited states of the complexes were further calculated, with the data summarized in Table S3. The first excited state of the singlet (S1) of Al(3-HF)_2_ was located at 353.96 nm, which was mainly composed of the transition from HOMO to LUMO (54.0%) and the transition from HOMO-1 to LUMO+1 (29.3%). Because of the heavy atom effect caused by the coordination of aluminum atoms, the S1 of Al(3-HF)_2_ was allowed to transfer to the triplet first excited state (T1; 484.40 nm) through the intersystem crossing, which consists mainly of transitions from HOMO to LUMO (96.2%). In addition, the S1 of Al(DCHQ)_3_ was located at 453.99 nm, which was mainly composed of the transition from HOMO to LUMO (69.0%). Interestingly, its T1 (534.43 nm) was also mainly composed of the transition from HOMO to LUMO (95.2%). The hole and electron distributions of S1 and T1 transitions of Al(3-HF)_2_ and Al(DCHQ)_3_ are shown in Figures S10 and S11, respectively. In summary, the consistency between simulations and experiments further elucidated the mechanism of luminescence.

### 3.4. Cyan Emission Tuning

**Figure 6:**
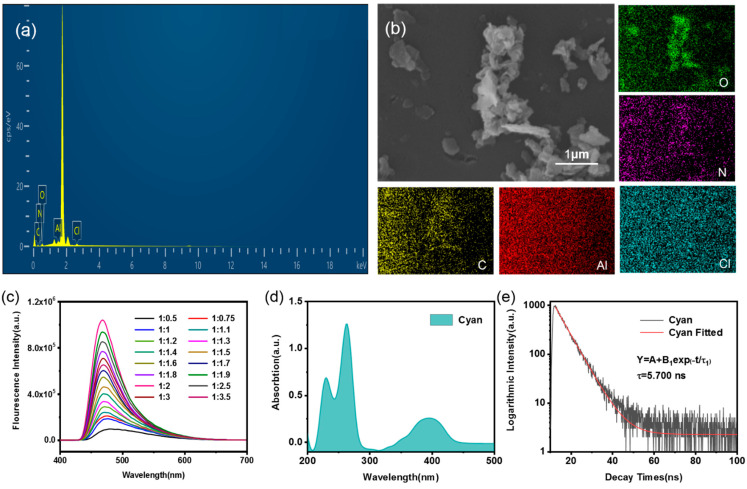
(**a**) EDS spectrum, (**b**) SEM image and EDS elemental mapping of the AHDAs with cyan light, (**c**) emission spectra of the mixture of Al(3-HF)_2_ and Al(DCHQ)_3_ solutions with varying volume ratios, (**d**) UV–VIS absorption spectrum of CLS, and (**e**) fitting time-resolved fluorescence decay curve of CLS.

To tune the cyan emission, a solution mixture of Al(3-HF)_2_ and Al(DCHQ)_3_ was prepared in the ratio 2:1 (Al(3-HF)_2_:Al(DCHQ)_3_). The EDS spectrum and SEM images of AHDAs are shown in Figure 6. The elements C, Al, O, N, Cl, and Si were detected (Figure 6a). The Si peak was attributed to the monocrystalline silicon substrate. The SEM images in Figure 6b show that the irregular aggregates were distributed on the substrate. To further investigate the distribution of each element, the elemental mapping images were recorded, as shown in Figure 6b. Clearly, each element was evenly distributed over the observed area.

The XPS data of CLM can provide a more detailed understanding of the mixing behavior between the two complexes. The binding energies of CLM to both complexes were consistent. As shown in Figure S12, the binding energy of Al 2p in CLM was 73.55 eV, that of O 1s was 531.5 eV, and that of N1s was 405.9 eV. Compared to the complexes, the binding energies of the elements in the CLM remained unchanged or changed little. In contrast, compared to metal salts and ligands, the binding energies of each element in CLM significantly changed. This is not only strong evidence for the formation of aluminum complexes but also confirmation of the mixing behavior between the two complexes.

Since cyan light can be obtained by the combinations of blue and green light, the solutions of the two complexes were mixed with varying volume ratios of aluminum (III) ions and two ligands, and the emission spectra of the mixed solutions were tested. As shown in Figure 6c, with the increase in the Al(3-HF)_2_ solution, the emission peak of the obtained mixed solution blue-shifted. Until the volume ratio (Al(DCHQ)_3_:Al(3-HF)_2_) was 1:2, the emission spectrum of the mixed solution shifted to 470 nm, having excellent fluorescence intensity. The mixed solution prepared with this volume ratio was selected as the typical solution. The UV–VIS spectrum is shown in Figure 6d for further investigation of photophysical properties. The first absorption band appeared at 210–290 nm, with peaks at 230 and 264 nm. Meanwhile, the second band appeared between 340 and 440 nm, with a peak at 396 nm. This result indicates that cyan emission can be obtained from the combination of blue and green light. Therefore, cyan luminescence is considered the result of spectral modulations.

To study the influence of aluminum (III) ion concentration, we diluted the standard solutions to different low-concentration samples. The excitation and emission spectra of these solutions of cyan are shown in Figure S13a,b. With dilution, the fluorescence intensities decreased gradually. As shown in Figure 6e, the fluorescence lifetime of cyan light was fitted to a single-exponential decay function, and the calculated fluorescence lifetime was 5.700 ns. The CIE coordinate points in Figure S14 show that this emission wavelength is in the cyan area. The coordinates of the blue light, green light, and cyan light are plotted in the same graph. The cyan light lies between the blue and green points, which proves that cyan light is made by mixing blue light and green light. This also shows that cyan light is the result of spectral modulations.

### 3.5. Selective Sensitivity to Different Metal Ions with Either Strong Enhancement or Quenching Effects

**Figure 7:**
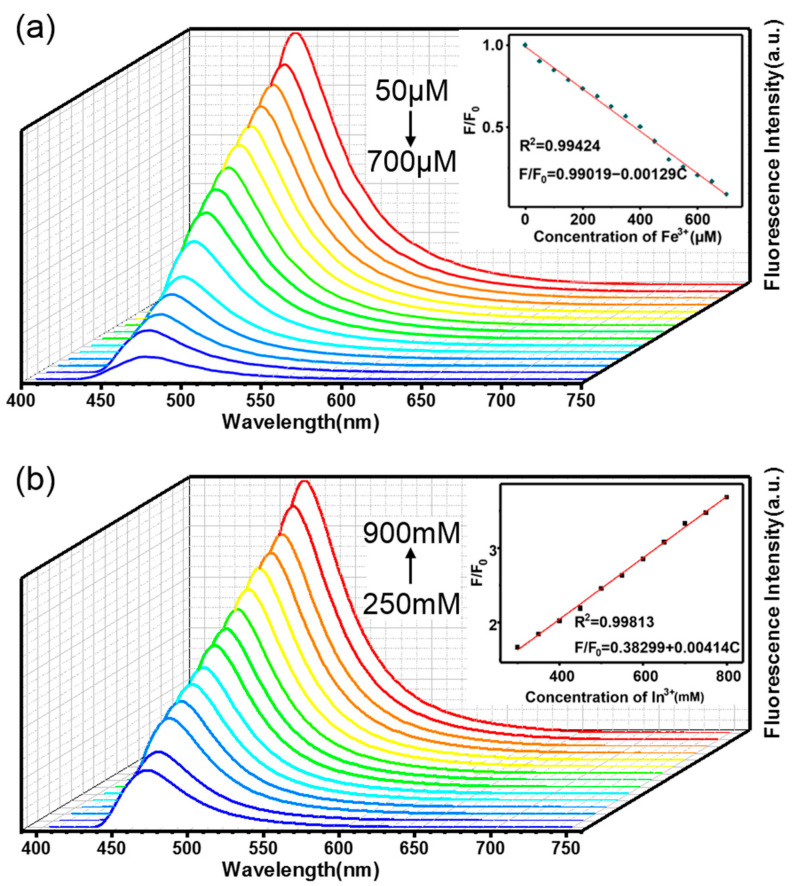
(**a**) Fluorescence intensity of CLS containing different concentrations of Fe^3+^ solutions (inset: linear relationship of the fluorescence intensity and Fe^3+^ concentration) and (**b**) fluorescence intensity of CLS with different concentration of In^3+^ solutions (inset: linear relationship of the fluorescence intensity and In^3+^ concentration).

Fe^3+^ deficiency in the human body affects normal immune function [33]. This is one important application for Fe^3+^ sensing. We designed experiments to verify the sensitivity of CLS to Fe^3+^ and investigated the effect of Fe^3+^ solutions (50–700 μM) to quench cyan fluorescence. Figure 7a demonstrates the quenching behavior, and the linear relationship between Fe^3+^ concentration and fluorescence intensity is plotted in the inset picture. The linear fit obtained was F/F_0_ = 0.99019 − 0.00129C (R^2^ = 0.99424). Here, F represents the fluorescence intensity of the solution after adding Fe^3+^, where F_0_ is the fluorescence intensity of the original solution, and C represents each concentration of the solution of Fe^3+^. Under this experimental condition, the limit of detection (LOD) was calculated according to LOD = 3δ/K (δ is the standard deviation of 10 blank samples, K is the slope of the linear coordinate curve), and the result was 4.79 μM [34]. Quenching may be due to the free-electron transition of the complexes to the outer electron orbits of Fe^3+^, which leads to a decrease in the fluorescence intensity of the solution added with Fe^3+^.

In^3+^ is used in several industrial applications, and sensitive detection of In^3+^ has practical significance. We found that In^3+^ in solution enhances the cyan emission of aluminum. Figure 7b shows the corresponding relationship between the concentration of In^3+^ and the fluorescence intensity of cyan emission of the aluminum complex. The linear relationship was expressed by F/F_0_ = 0.38299 + 0.00414C (R^2^ = 0.99813), as shown in the inset picture, and the meaning of each letter is the same as described before. Under this experimental condition, the LOD for In^3+^ was calculated to be 22.95 mM [34]. Since indium and aluminum are in the same group in the periodic table, it is considered that the exchange of ligands may have occurred. That is, some indium complexes may be formed, thus increasing the fluorescence intensity of the solution. Substances that can be used to detect the content of Fe^3+^ have been widely reported, but there are few studies on the detection of In^3+^, and substances that can be used for the detection of two ions at the same time have not been found. Therefore, CLS from single aluminum (III) ion centers is an excellent material with potential advantages.

To demonstrate the selectivity of the aluminum complexes for Fe^3+^ and In^3+^ detection, a group of different ions at the same concentration used before were compared in fluorescence measurement experiments. The different effects of the metal ions are presented in Figure S15. The solution of Al(3-HF)_2_ was the least sensitive to these metal ions, as shown in Figure S15a. In this process, the solutions with Fe^2+^, Cu^2+^, and Fe^3+^ were detected to have a slight quenching phenomenon, while the solutions with Zn^3+^, Ni^3+^, and Eu^3+^ showed enhancement of fluorescence intensity. When observing the behaviors of metal ions in the solution of Al(DCHQ)_3_ (Figure S15b), Fe^3+^, Cu^2+^, Zn^2+^, and Co^2+^ were found to have a slight quenching effect on the original solution, and the addition of Mg^2+^, Cr^3+^, and In^3+^ enhanced the fluorescence intensity. Interestingly, compared to the two complex solutions, combined Al(3-HF)_2_ and Al(DCHQ)_3_ with cyan fluorescence is more sensitive to those metal ions, as shown in Figure S15c. Among them, the fluorescence intensity of the solution containing Fe^3+^, Cu^2+^, and Cr^3+^ showed a significant decrease, while In^3+^, Ca^2+^, Mg^2+^, Mn^2+^, and Fe^2+^ were observed to enhance the fluorescence intensity of the original solution.

To test the reusability of CLS for ions, we designed two sets of experiments. First, we added 1 mM-1 M ethylenediaminetetraacetic acid (EDTA) to CLS in the presence of Fe^3+^, and it was found that the fluorescence gradually recovered, as shown in Figure S16a, and the fluorescence intensity finally recovered was 48.2%, as before. In addition, the presence of Fe^3+^ and functional group chelation on the surface of complexes led to quenching. Similarly, we added 1 mM-1 M EDTA to CLS in the presence of In^3+^ and found a slight decrease in the fluorescence intensity, as shown in Figure S16b.

## 4. Conclusions

In summary, multi-color emissions from aluminum complexes are innovatively realized by ligand tuning. 3-HF and DCHQ were selected to synthesize two aluminum complexes capable of emitting in the green and blue regions. The complexation was analyzed by FT-IR and XPS, and the photophysical properties of the complexes were improved after the reaction. The structural details and photophysical properties of the complexes were further confirmed by DFT simulations. Among them, ILCT exists in Al(3-HF)_2_ molecules, while LLCT exists in Al(DCHQ)_3_ molecules. The two complex solutions were mixed, and broadband cyan emission at 470 nm was achieved by adjusting the ratio of aluminum (III) ions and the two ligands. The uniform distribution of each element in mixtures with cyan light was verified by EDS mapping. Cyan fluorescent mixtures obtained by ligand tuning have high QYs and extremely high fluorescence intensity. In addition, the mixtures have better sensitivity to metal ions and an obvious linear relationship for the detection of Fe^3+^ and In^3+^. Some metal ions, including Cu^2+^ and Cr^3+^, have a quenching effect on cyan light, while Mn^2+^, Ca^2+^, Mg^2+^, and Fe^2+^ can enhance its fluorescence intensity to different degrees. This result not only proves that CLS could be a promising sensor material to detect metal ions but also that CLS has potential applications in the preparation of high-intensity cyan light from double-metal-ion complexes.

## Data Availability

The data presented in this study are available on request from the corresponding author after obtaining permission from an authorized person.

## Supplementary Materials

The following supporting information can be downloaded at: https://www.mdpi.com/article/10.3390/ma15155199/s1, Figure S1: Chemical structures of ligands; Figure S2: XPS survey of Al(NO_3_)_3_, two ligands and two complexes; Figure S3: XPS spectra of C 1s in Al(NO_3_)_3_ and complexes; Figure S4: Emission spectra of different aluminum complexes with blue fluorescence; Figure S5: Fitting time-resolved fluorescence decay curves of three aluminum complexes with blue fluorescence; Figure S6: CIE chromaticity coordinates of blue fluorescence emission; Figure S7: Emission spectra of different aluminum complexes with green fluorescence; Figure S8: Fitting time-resolved fluorescence decay curves of three aluminum complexes with green fluorescence; Figure S9: CIE chromaticity coordinates of green fluorescence emission; Figure S10: The hole and electron distributions of S1 and T1 transition of Al(3-HF)_2_; Figure S11: The hole and electron distributions of S1 and T1 transition of Al(DCHQ)_3_; Figure S12: XPS spectra of (**a**) Al 2p, (**b**) O 1s, (**c**) N 1s in CLM; Figure S13: Excitation and emission spectra of cyan light solutions with the different concentration of aluminum (III) ions; Figure S14: CIE chromaticity coordinates of blue, green and cyan; Figure S15: Comparison of fluorescence efficiency by Al(3-HF)_2_, Al(DCHQ)_3_, and cyan fluorescence solutions with 14 kinds of metal ions at the same concentration; Figure S16: Fluorescence intensity of CLS containing (**a**)Fe^3+^, (**b**)In^3+^ with 1 mM-1 M EDTA added. Table S1: Comparison of quantum yield and fluorescence lifetime of aluminum complexes with different ligands that produce blue fluorescence; Table S2: Comparison of quantum yield and fluorescence lifetime of aluminum complexes with different ligands that produce green fluorescence; Table S3: The calculated wavelength, oscillator strength and compositions of major transitions of Al(3-HF)_2_; Table S4: The calculated wavelength, oscillator strength and compositions of major transitions of Al(DCHQ)_3_; Table S5: Emission peaks of mixtures with different volume ratios of Al(3-HF)_2_ and Al(DCHQ)_3_.
