# Supplementary material for: Ligand-Tuned Multi-Color Luminescence of Single Aluminum (III) Ion Atomic Centers and Their Selective Sensitivity to Different Metal Ions
Source: Materials (Basel). 2022 Jul 27;15(15):5199. doi: 10.3390/ma15155199 (PMC9370060; doi:10.3390/ma15155199)
Supplement: Supplementary file 1 [file materials-15-05199-s001.zip › materials-1811515-supplementary.pdf]

Supporting information for:

Article

Ligand-Tuned Multi-Color Luminescence of Single Aluminum (III) Ion Atomic Centers and Their Selective Sensitivity to Different Metal Ions

Qian Wang [1,†], Longlong Li [1,†], Qinglin Tang [1], Jin Liu [1], Yao Wang [1], Jiuxing Wang [1], Matt J. Kipper [2]**, Haijiao Xie [3], Laurence A. Belfiore [1,2] and Jianguo Tang [1,*]**

1 Institute of Hybrid Materials, National Center of International Joint Research for Hybrid Materials Technology, National Base of International Sci. & Tech. Cooperation on Hybrid Materials, Qingdao University, 308 Ningxia Road, Qingdao 266071, China; reamiss@163.com (Q.W.); babylove8856@126.com (L.L.); a15666920912@163.com (Q.T.); liujin0620@126.com (J.L.); wangyaoqdu@126.com (Y.W.); jiuxingwang@qdu.edu.cn (J.W.); belfiore@engr.colostate.edu (L.A.B.)

2 Department of Chemical and Biological Engineering, Colorado State University, Fort Collins, CO 80523, USA; matthew.kipper@colostate.edu

3 Hangzhou Yanqu Information Technology Co., Ltd., Y2, 2nd Floor, Building 2, Xixi Legu Creative Pioneering Park, No. 712 Wen'er West Road, Xihu District, Hangzhou 310003, China; xiehaijiao@shiyanjia.com

* Correspondence: tang@qdu.edu.cn; Tel.: Tel.: +86-137-9180-1659

† These authors contributed equally to this work.

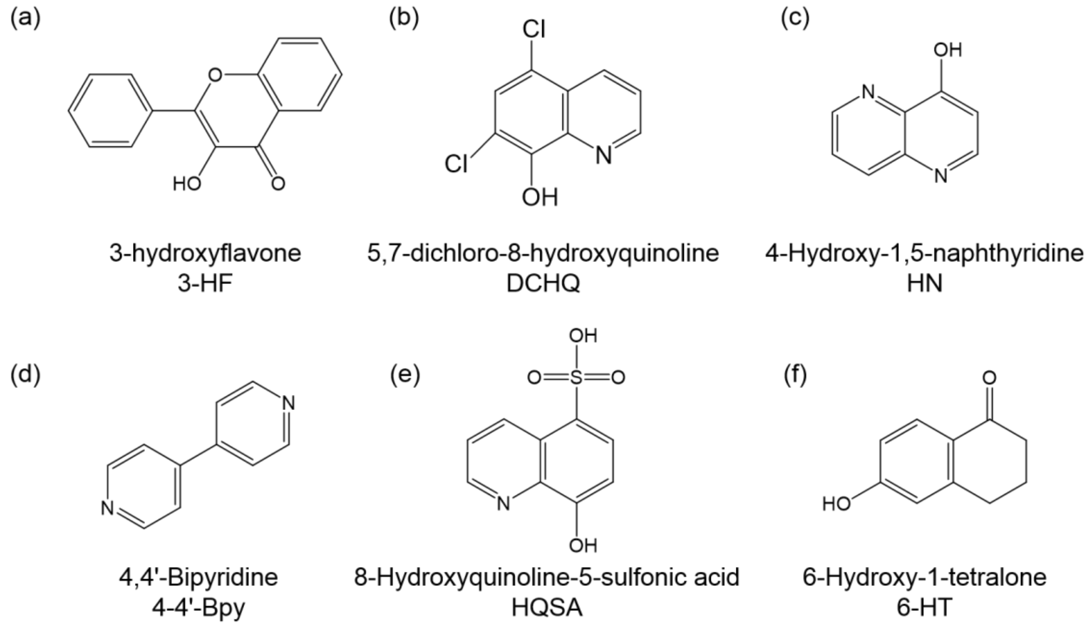

Figure S1. Chemical structures of ligands.

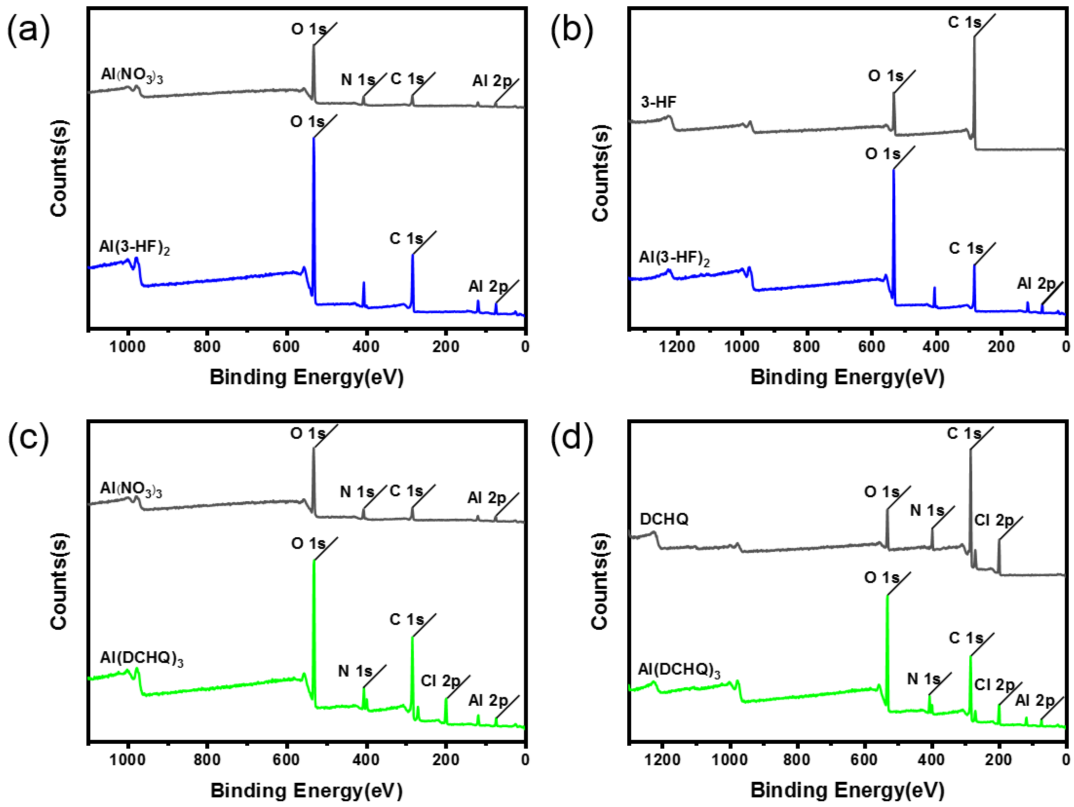

Figure S2. XPS survey of (a) $Al(NO_3)_3$ and $Al(3\text{-}HF)_2$, (b)3-HF and $Al(3\text{-}HF)_2$, (c) $Al(NO_3)_3$ and $Al(DCHQ)_3$, (d) DCHQ and $Al(DCHQ)_3$.

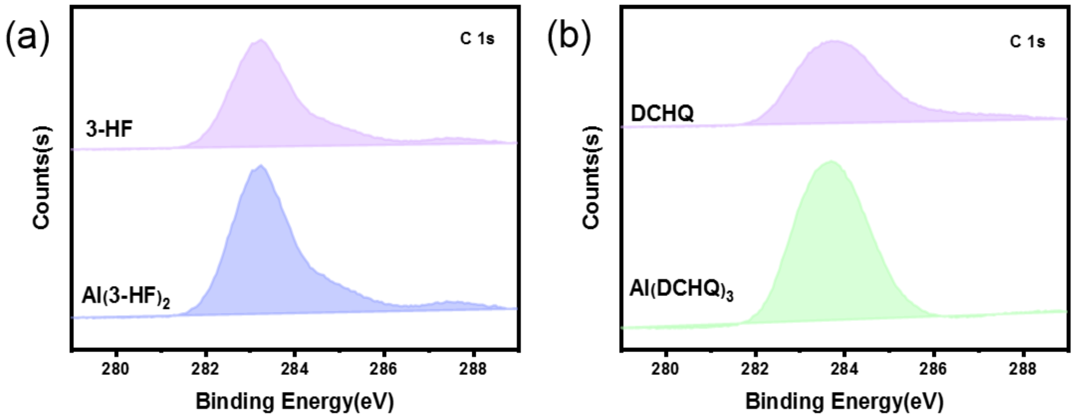

Figure S3. XPS spectra of C 1s in $Al(NO_3)_3$ and complexes.

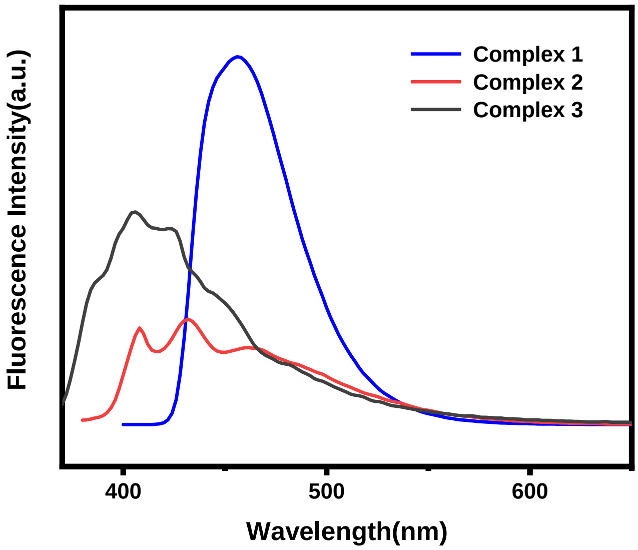

Figure S4. Comparison of the fluorescence intensity of different aluminum complexes with blue fluorescence.

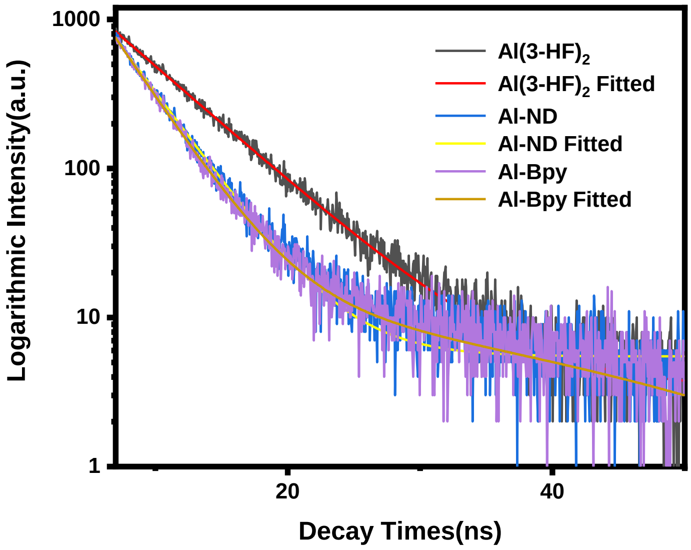

Figure S5. Fitting time-resolved fluorescence decay curves of three aluminum complexes with blue fluorescence.

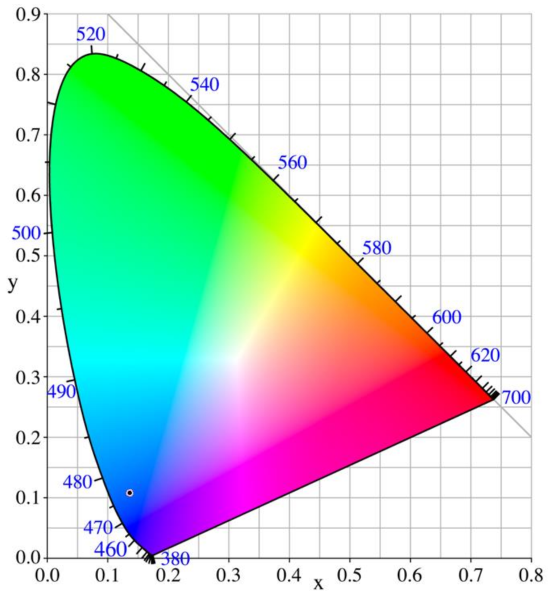

Figure S6. CIE chromaticity coordinates of blue fluorescence emission.

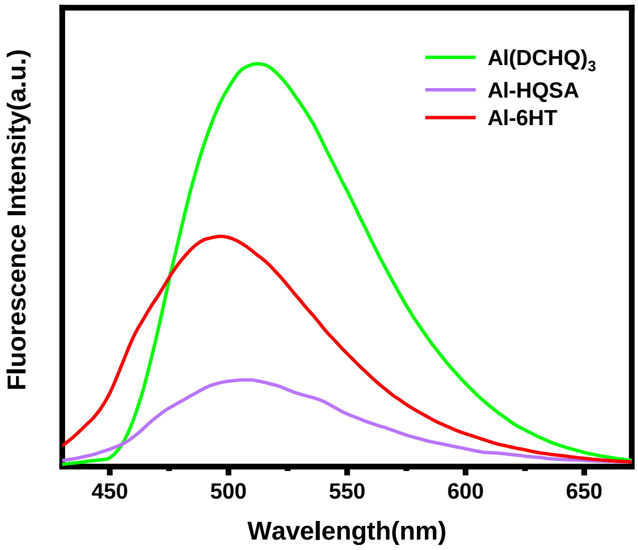

Figure S7. Comparison of the fluorescence intensity of different aluminum complexes with green fluorescence.

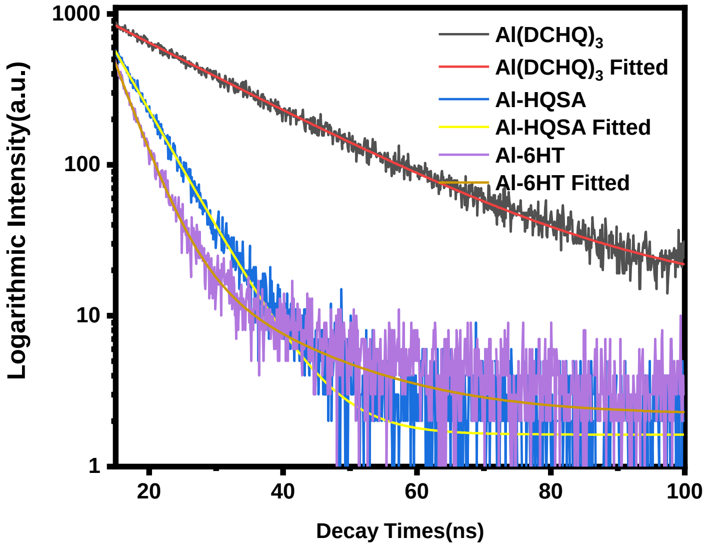

Figure S8. Fitting time-resolved fluorescence decay curves of three aluminum complexes with green fluorescence.

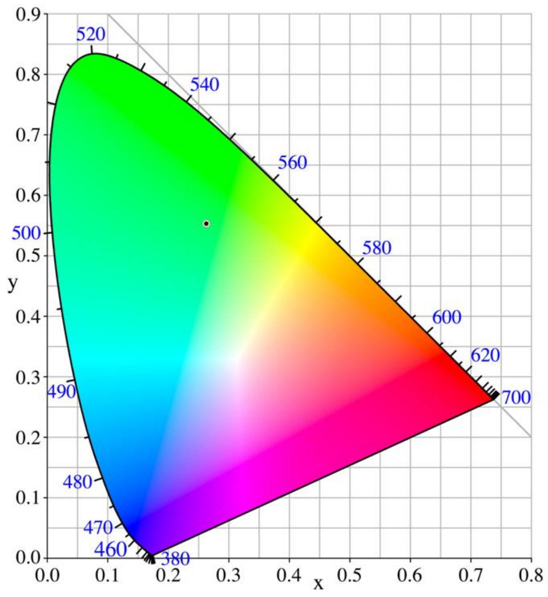

Figure S9. CIE chromaticity coordinates of green fluorescence emission.

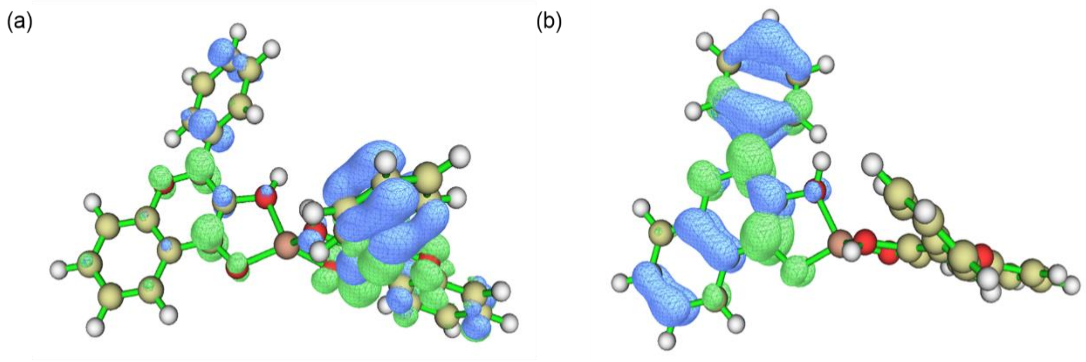

Figure S10. The hole and electron distributions of (a) S1 and (b) T1 transition of $Al(3\text{-}HF)_2$. Blue and green isosurfaces represent hole and electron distributions, respectively.

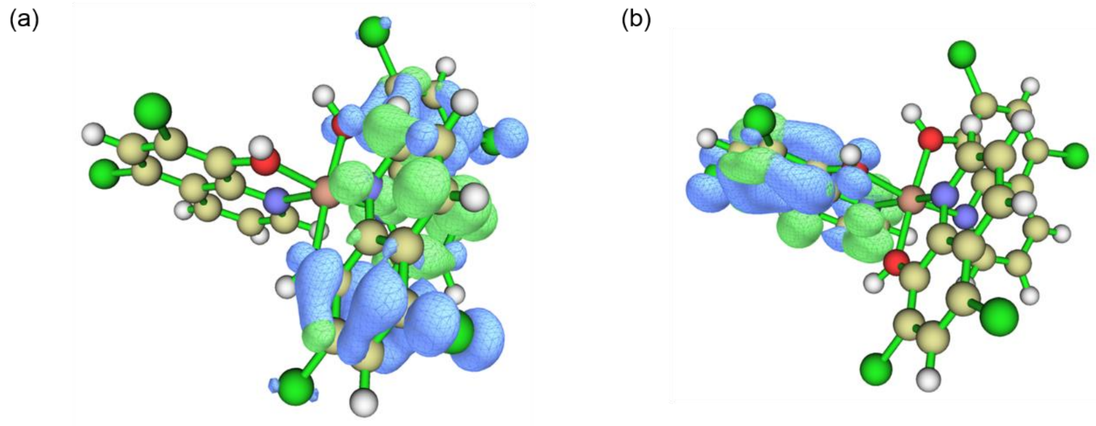

Figure S11. The hole and electron distributions of (a) S1 and (b) T1 transition of Al(DCHQ)$_3$. Blue and green isosurfaces represent hole and electron distributions, respectively.

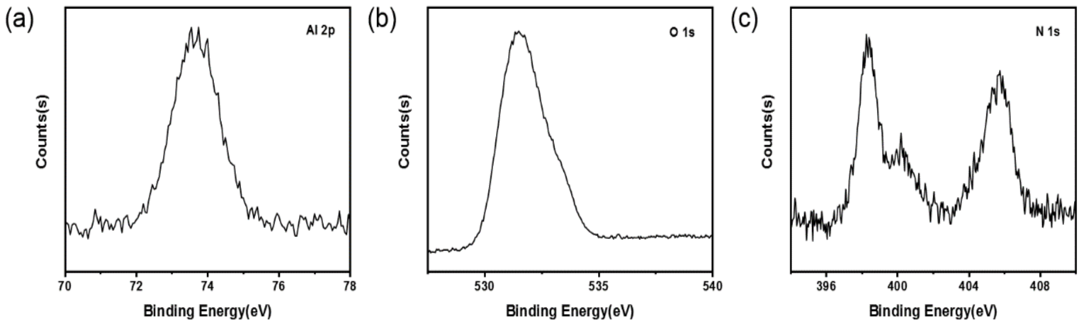

Figure S12. XPS spectra of (a) Al 2p, (b) O 1s, (c) N 1s in CLM.

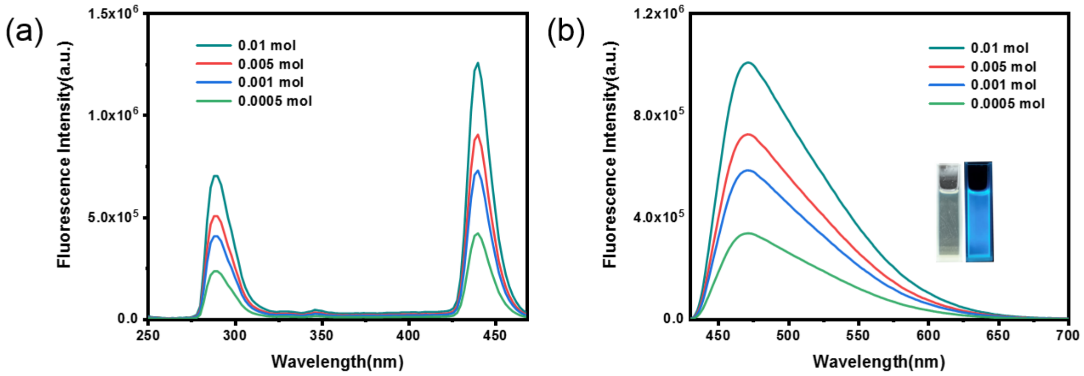

Figure S13. Comparison of (a) excitation spectra and (b) emission spectra of cyan light solutions with the different concentration of aluminum (III) ions.

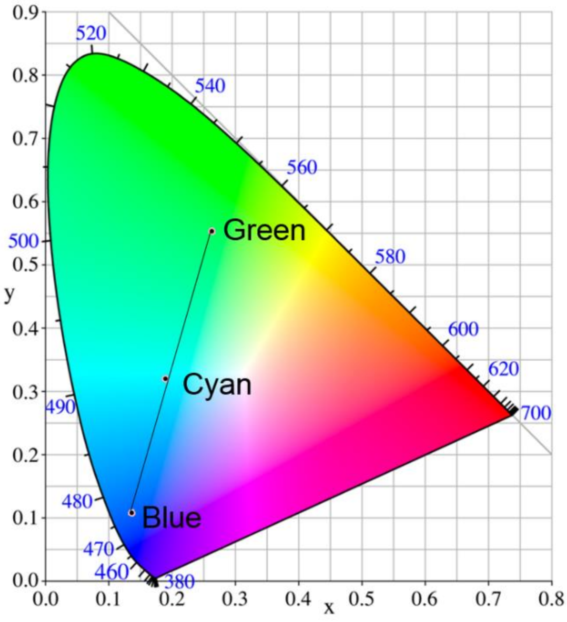

Figure S14. CIE chromaticity coordinates of blue, green and cyan.

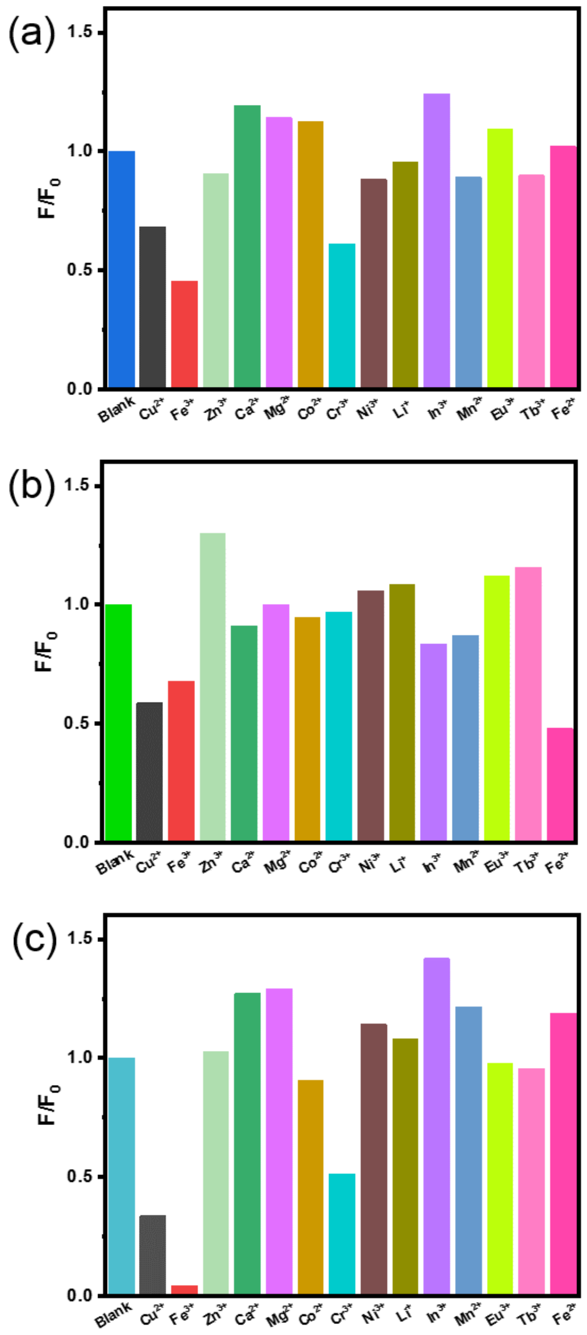

Figure S15. Comparison of fluorescence efficiency by (a) Al(3-HF)$_2$, (b) Al(DCHQ)$_3$, and (c) cyan fluorescence solutions with 14 kinds of metal ions at the same concentration.

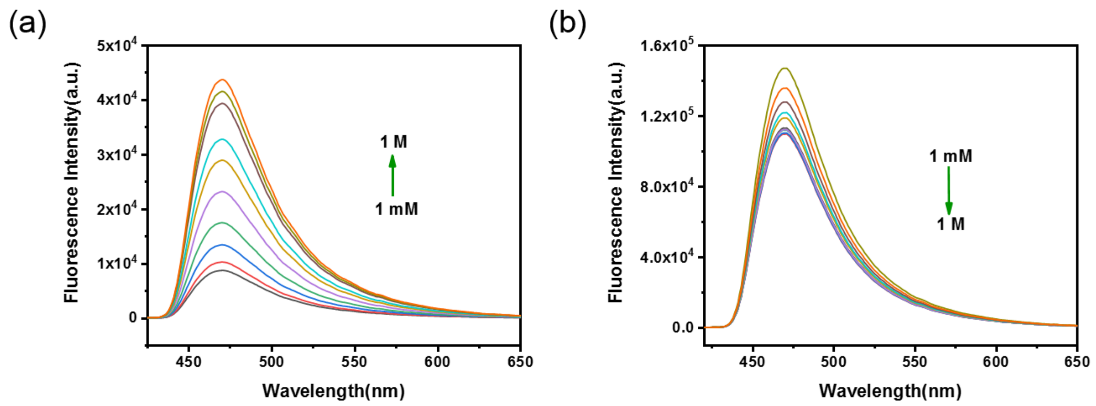

Figure S16. Fluorescence intensity of CLS containing (a)Fe^{3+}, (b)In^{3+} with 1 mM-1 M EDTA added.

Table S1. Comparison of quantum yield and fluorescence lifetime of aluminum complexes with different ligands that produce blue fluorescence.

Sample	τ_1(ns)	τ_2(ns)	B_1	B_2	χ^2	τ_{ave}(ns)	QY (%)
3-HF	5.606	-	944.811	-	1.075	5.606	29.42%
ND	3.573	-	943.732	-	1.363	3.573	11.41%
4,4'-Bpy	3.242	40.19	1045.743	21.155	1.264	10.650	8.57%

Table S2. Comparison of quantum yield and fluorescence lifetime of aluminum complexes with different ligands that produce green fluorescence.

Ligands	τ_1(ns)	τ_2(ns)	B_1	B_2	χ^2	τ_{ave}(ns)	QY (%)
DCHQ	18.81	-	950.582	-	1.070	18.810	37.00%
HQSA	5.562	-	976.010	-	1.051	5.562	17.38%
6-HT	3.474	14.83	978.723	32.824	1.127	4.897	7.05%

Table S3. The calculated wavelength, oscillator strength and compositions of major transitions of $Al(3\text{-}HF)_2$.

Wavelength (nm)	Osc. Strength(*f*)	Major contribution
353.96(S1)	0.5301	HOMO→LUMO (54.0%)
		HOMO-1→LUMO+1 (29.3%)
484.40(T1)	0.0000	HOMO→LUMO (96.2%)

Table S4. The calculated wavelength, oscillator strength and compositions of major transitions of $Al(DCHQ)_3$.

Wavelength (nm)	Osc. Strength(*f*)	Major contribution
453.99 (S1)	0.1586	HOMO→LUMO (69.0%)
534.43 (T1)	0.0000	HOMO→LUMO (95.2%)

Table S5. Emission peaks of mixtures with different volume ratios of $Al(3\text{-}HF)_2$ and $Al(DCHQ)_3$.

Volume ratio($Al(DCHQ)_3$:$Al(3\text{-}HF)_2$)	Emission peak(nm)
1:0.5	482
1:0.75	480
1:1	478
1:1.1	478
1:1.2	478
1:1.3	476
1:1.4	476
1:1.5	474
1:1.6	474
1:1.7	472
1:1.8	470
1:1.9	470
1:2	470
1:2.5	468
1:3	468
1:3.5	468
